# From OCD Traps to Transformative Dialogue: Managing a Case of OCD and Body Dysmorphic Disorder with Dramatised Socratic Dialogue

**DOI:** 10.1192/j.eurpsy.2025.1718

**Published:** 2025-08-26

**Authors:** F. Raffone, A. Russo, A. Capasso, F. Mancini, A. M. Saliani

**Affiliations:** 1 Department of Mental Health, Asl Napoli 1 Centro; 2 Department of Mental Health, Asl Napoli 3 Sud, Naples; 3 Scuole di Psicoterapia Cognitiva APC-AIPC-SPC-SICC; 4 Scuole di Psicoterapia Cognitiva APC-SPC-AIPC-SICC , Rome, Italy

## Abstract

**Introduction:**

Obsessive-compulsive disorder (OCD) and body dysmorphic disorder (BDD) often co-occur, creating complex symptom profiles and requiring multifaceted treatment approaches. OCD can lead to intense self-monitoring and distressing obsessive-compulsive behaviours, while BDD contributes to a distorted self-image, exacerbating feelings of inadequacy and shame. This case study explores the use of a metacognitive approach using Dramatised Socratic Dialogue (DSD) in the treatment of a patient with severe OCD and BDD, focusing on relational challenges, pervasive shame and self-criticism.

**Objectives:**

To evaluate the effectiveness of dramatised Socratic dialogue and exposure with response prevention (E/RP) in the treatment of a complex case of OCD and BDD. Specifically, to address intrusive self-criticism, enhance the therapeutic alliance, and reduce shame-related behaviours.

**Methods:**

A 33-year-old man with a long history of OCD and BDD symptoms, including excessive mirror checking and social avoidance, was assessed using the MMPI-2, PID-5, MADRS, STAY-1 and 2, and Y-BOCS, confirming OCD, BDD, and major depressive disorder. Treatment included establishing a strong therapeutic alliance, psicoeducation, E/RP and DSD targeting persistent self-criticism. After an initial phase, interventions focused on reducing compulsive behaviours and promoting self-acceptance.

**Results:**

Initial E/RP led to symptom improvement but maintained a sense of control that limited full therapeutic progress. DSD successfully reduced self-critical dialogue and addressed shame and self-perceived social unacceptability, although it temporarily disrupted the therapeutic alliance. Subsequent reintegration of E/RP alongside DSD facilitated substantial reductions in OCD and BDD symptoms, with the patient reporting increased mood stability and reduced social avoidance.

**Conclusions:**

This case highlights the benefits of integrating dramatised Socratic dialogue with traditional CBT methods such as E/RP to address OCD and BDD symptoms where shame and self-criticism are significant. DSD proved effective in reframing negative self-talk, breaking cycles of self-criticism and supporting long-term symptom reduction. This approach shows promise for treating complex cases involving intense feelings of inadequacy and shame.

**Disclosure of Interest:**

None Declared

